# Differentiating cyclopropane fatty acids to support milk authenticity through GC–MS and NMR spectroscopy

**DOI:** 10.1016/j.fochx.2025.103033

**Published:** 2025-09-16

**Authors:** Gian Marco Riccio, Dilek Eltemur, Federico Fava, Demian Martini-Lösch, Giovanni Peratoner, Elena Venir, Daniela Eisenstecken, Peter Robatscher, Matteo Scampicchio, Michael Oberhuber, Alberto Ceccon

**Affiliations:** a Laimburg Research Centre, Laimburg 6 – Pfatten (Vadena), 39040 Auer (Ora), BZ, Italy; b Faculty of Agricultural, Environmental and Food Sciences, Free University of Bozen-Bolzano, Piazza Università 5, 39100 Bozen-Bolzano, Italy

**Keywords:** DHSA, LBA, Solution NMR spectroscopy, Gas chromatography, Mass spectrometry, Haymilk, Molecular markers

## Abstract

Cyclopropane fatty acids (CPFAs), are emerging markers for food authentication in premium dairy products like *Parmigiano Reggiano* and *Haymilk*, where silage feeding is prohibited. This study focuses on two CPFAs—dihydrosterculic acid (DHSA) and lactobacillic acid (LBA)—in milk from cows fed diets with or without silage. Using both NMR spectroscopy and a refined GC–MS method, we quantified CPFAs and assessed their potential as dietary markers. NMR enabled rapid, non-destructive screening via *cis*-methylene proton signals, whereas GC–MS provided accurate quantification and separation of DHSA and LBA. DHSA levels were ∼ 3-fold higher in maize-silage-fed cows compared to non-silage-fed cows, making DHSA, or the DHSA/LBA ratio, robust markers of feeding regime. In contrast, LBA remained stable across diets and was unexpectedly detected in Haymilk, suggesting alternative metabolic origins. These results demonstrate the value of combining NMR and GC–MS for accurate CPFA quantification and provide new insights into their role in dairy authentication.

## Highlights

• CPFAs in milk indicate the presence of silage in cow's ration.
• NMR analysis is suitable for preliminary screening of the overall CPFAs content.
• Improved GC–MS analysis allows separate quantification of DHSA and LBA.
• LBA occurs in all milk samples with lower concentration in Haymilk.
• Differences between milk samples are primarily driven by DHSA content.

## Graphical abstract

**Figure:**
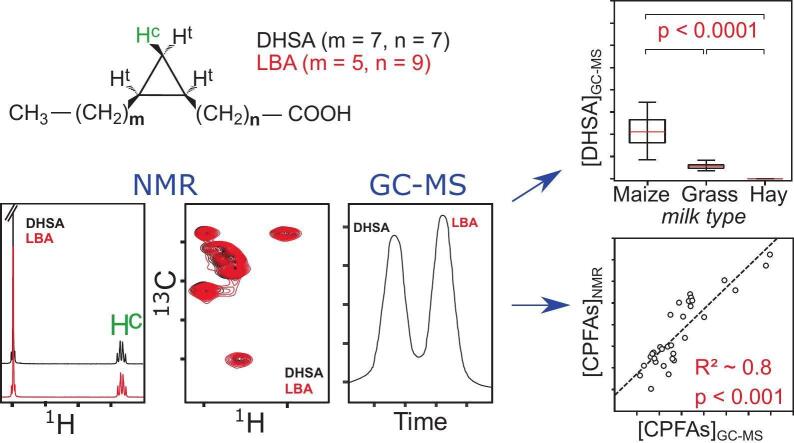

## 1 Introduction

Cyclopropane fatty acids (CPFAs) are unusual fatty acids containing a three‑carbon ring along the fatty acid chain. They are mainly found in bacterial membranes of both Gram-positive and Gram-negative species, often as a response to stress conditions (Caligiani & Lolli, 2018; Moore & Floss, 1999).

CPFAs have also been detected in animal-derived foods, including dairy and meat products, where their presence is associated with the use of maize silage in livestock feeding (Fava et al., 2025; Imperiale et al., 2021; Lolli et al., 2021a; Marseglia et al., 2013). Recently, the presence of CPFAs in milk and dairy products has gained significance in the realm of food authentication. For instance, products like Parmigiano Reggiano cheese (PDO) and Haymilk, where silage usage is forbidden (EU 1151/12, EU 2016/304), are expected to be devoid of CPFAs (Caligiani et al., 2016).

Given the high market value of such products and the growing concern over food fraud, CPFAs are recognized as molecular and quality markers in dairy products (Caligiani et al., 2016; Marseglia et al., 2013). At the same time, their biological role and potential effects on human health remain poorly understood (Fox et al., 2024; Lolli et al., 2019; Quach et al., 2019). This makes the development of accurate analytical methods essential, both to improve detection and to gain insights into their origin.

Traditionally, CPFAs have been quantified using gas chromatography coupled with mass spectrometry (GC–MS), which offers high sensitivity and specificity (Caligiani et al., 2016; Ecker et al., 2012; Lolli et al., 2022). However, GC–MS involves time-intensive sample derivatization, significant solvent use, and risks of interference from by-products. Given these limitations, nuclear magnetic resonance (NMR) spectroscopy has recently emerged as a promising alternative, offering faster analysis with minimal sample preparation and avoids the need for derivatization (Eltemur et al., 2023; Iannone et al., 2024; Lolli et al., 2018). This method involves simple fat dissolution in deuterated chloroform and benefits from increased sensitivity due to modern pulse sequences and decoupling strategy as recently shown (Eltemur et al., 2023).

Main CPFAs found in dairy products are dihydrosterculic acid (*cis*-9,10-methyleneoctadecanoic, DHSA) and lactobacillic acid (*cis*-11,12-methyleneoctadecanoic, LBA) (Caligiani et al., 2014). In cow milk and several dairy products, CPFAs were detected in significant amounts, up to 10^3^ mg kg^−1^ of extracted milk fat, corresponding to ∼0.1 % w/w of the total fatty acid contents (Caligiani et al., 2014; Imperiale et al., 2021; Lolli et al., 2020).

Previous studies that listed the content of CPFAs have primarily reported them without differentiation of DHSA and LBA in dairy products (Caligiani et al., 2014; Fava et al., 2025; Iannone et al., 2024; Imperiale et al., 2021; Marseglia et al., 2013). This approach is largely driven by practical considerations. Detecting LBA can be challenging, particularly when it occurs in smaller quantities, or its signal is indistinguishable from the other CPFA (Caligiani et al., 2016; Lolli et al., 2021a; Lolli et al., 2018, 2020, 2022; Lolli et al., 2021b; Montanari et al., 2010).

However, since LBA and DHSA are derived from different precursors – C18:1 n11 (vaccenic acid) and C18:1 n9 (oleic acid), synthesized via anaerobic and aerobic pathways respectively (Montanari et al., 2010) - a detailed quantitative assessment of their absolute and relative concentrations is expected to provide better insights into these metabolic processes, particularly in response to stress conditions. Moreover, this may lead to mechanistic insights into the metabolic dynamics and the environmental or dietary factors influencing milk composition (Fava et al., 2025; Imperiale et al., 2021). Such precision is essential for enhancing authenticity control, thereby supporting the integrity and transparency of the dairy supply chain.

This study aims to fill this knowledge gap by investigating the prevalence of DHSA and LBA in dairy products. We employed a refined GC–MS method that enables improved separation and accurate identification of these compounds, overcoming limitations of earlier approaches. By integrating GC–MS with NMR spectroscopy, we analyzed how different feeding regimes influence the CPFA profile in milk. Importantly, we specifically aim at the separate quantification of DHSA and LBA in milk, providing a distinctive compositional feature relevant for authentication and characterization of this traditional product.

## 2 Material and methods

### 2.1 Materials

(±)-cis-9,10-methyleneoctadecanoic acid (DHSA) (CAS N. 5711-28-4, Larodan, Solna, Sweden), (±)-cis-11,12-methyleneoctadecanoic acid (LBA) (CAS N. 19,625–10-6, Larodan, Solna, Sweden), methyl cis-9,10-methyleneoctadecanoate (methylated-DHSA) (CAS N. 3971-54-8, Larodan, Solna, Sweden), methyl cis-11,12-Methyleneoctadecanoate (methylated-LBA) (CAS N. 5965-63-9, Larodan, Solna, Sweden), acetophenone-d8 (CAS N. 19,547–00-3, Sigma Aldrich Saint Louis, MO, USA), deuterated chloroform (CDCl₃, 0.03 *v*/v % TMS as an internal standard, CAS N. 865–49-6, >98 % D, Sigma-Aldrich, Saint Louis, MO, USA) were used. All other used chemicals (solvents, standards, and reagents) were of analytical grade.

### 2.2 Milk sampling

This study analyzed three types of milk: M-milk (from cows fed a diet including maize silage with no other fermented feed), G-milk (from cows fed a diet including grass silage with no other fermented feed), and H-milk (Haymilk). For the latter, cows were fed in accordance with EU Regulation 2016/304 for Haymilk production, which stipulates that no fermented fodder is used, and roughage must constitute at least 75 % of the dry matter in their annual diet. Sampling campaigns were conducted as previously described by Fava et al. (2025). Fresh raw bulk milk samples were collected from dairy farms in South Tyrol (NE Italy), located at altitudes ranging from 616 m to 1404 m above sea level (a.s.l.). To account for seasonal variations, milk samples were collected during three periods: Winter 2019–2020 (w-19.20), Winter 2020–2021 (w-20.21), and Summer 2020 (s-20). At each sampling event, 270 mL of milk was collected from the same tank, with 30 mL reserved for CPFA analysis. Samples were transported at 4 °C for a maximum of 2 h and stored at −80 °C until analysis. This study builds upon the previous research reported by Imperiale et al. (Imperiale et al., 2021), who used a targeted approach to measure DHSA in samples collected exclusively during Winter 2019–2020. Within a pool of 187 milk samples (37H-milk samples, 72 M-milk samples and 78 G-milk samples) preliminarily analyzed by GC–MS as described by Fava et al. (2025) the 10 samples of M-milk and G-milk with the highest CPFAs content respectively, as well as 10 randomly selected H-milk samples, were analyzed in this study for each sampling period. Due to the loss of one G-milk sample from the sampling period Winter 2019–2020 during the transesterification process, a total number of 89 samples were investigated.

### 2.3 Milk fat extraction for GC–MS and NMR analysis

The frozen milk samples were warmed in a water bath at 40 °C for 2 h. Fat extraction followed a revised procedure adapted from Feng et al. (Feng et al., 2004) For this process, 20 mL of milk was placed in a 50 mL plastic tube and centrifuged at 15,770 ×*g* for 30 min at 4 °C. After centrifugation, the upper fat layer was collected and transferred into a 15 mL plastic tube and stored overnight at −80 °C. The next day, the fat was dissolved in 10 mL of a 9:1 (v/v) n-pentane/methanol mixture. This mixture underwent several steps for thorough mixing and preparation: it was shaken vigorously for 2 min at room temperature, followed by a 5-min ultrasonic bath at 45 kHz. Next, it was agitated for 5 min using a MultiRotator (PTR-60, Grant Instruments, Royston, UK), and then shaken vigorously again for 2 min. The preparation concluded with a final centrifugation at 3220 ×*g* for 2 min at room temperature. The upper organic layer was carefully transferred into a tinted glass vial, and nitrogen gas was used to evaporate the solvent, leaving only the dry fat. This extracted fat was then stored at −80 °C until it was used for transesterification or the NMR analysis.

### 2.4 Transesterification of milk fat for GC–MS measurements

Transesterification was performed according to the method ISO 15884. (Caligiani et al., 2016) 100 (± 5) mg of milk fat was dissolved in 5 mL of heptane. Then, 0.2 mL of a 2 M KOH solution in dry methanol was added, and the solution was agitated briefly (30 s) to ensure thorough mixing. After an additional 5 min, 0.5 g of NaHSO_4_ was added to stop the reaction, and the mixture was agitated for another 30 s. The solution was subsequently centrifuged at 3220 ×*g* for 5 min at room temperature. The clear supernatant obtained (fatty acid methyl esters, FAMEs) was then used for GC–MS measurements.

### 2.5 GC–MS measurements

GC–MS analysis was performed using a GC MS-QP2010 SE (Shimadzu, Kyoto, Japan) equipped with an autosampler, a split/splitless injector and a single quadrupole mass spectrometer. For the analysis, 20 μL of the fatty acid methyl esters and 20 μL of Internal Standard (IS) (2 mg L^−1^ acetophenone-d8 in heptane) were diluted in 160 μL of heptane. From this solution, 1 μL was injected at 280 °C using a split ratio of 1:10 to optimize the detection of DHSA and LBA while preventing column overloading. The analytes were chromatographically separated using a low-polarity SLB-5 ms column (30 m length, 0.25 mm inner diameter, 0.25 μm film thickness) (Supelco, Bellefonte, PA, USA) with helium as the carrier gas at a flow rate of 1.3 mL min^−1^. As a difference to the method of (Montanari et al., 2010), we optimized the temperature ramp as follows: the GC oven temperature program was set as follows: 120 °C for 5 min, then increased to 180 °C at a rate of 3 °C min^−1^, held for 5 min, further increased to 200 °C at 0.5 °C min^−1^, and finally ramped up to 210 °C at 25 °C min^−1^. The transfer line temperature was 280 °C, and electron ionization (EI) at 70 eV was applied. The ion source temperature was 230 °C. All mass spectra were acquired in full scan (mass range 40–350 *m*/*z*) and in Selected Ion Monitoring (SIM) mode for improving the signal to noise ratio of IS, DHSA and LBA. Table S1 shows the list of quantifier and qualifiers ions for IS, DHSA and LBA. Quantification of both DHSA and LBA was conducted by comparing the area of the corresponding quantifier ion to that of the IS (Table S1). For GC–MS measurements, the limit of detection (LOD) and quantification (LOQ) for DHSA and LBA were determined from calibration curves (Fig. S1) which were obtained using transesterified complex mixture (obtained from H-milk samples assumed to be CPFAs-free) to account for matrix effect. The validation data for the method are as follows: LOD and LOQ are 13.7 and 41.5 mg kg^−1^ for DHSA and 11.7 and 35.3 mg kg^−1^ for LBA, respectively. The linear measurement range is from LOQ to 1500 mg kg^−1^, the recovery 101.5 % (0.2 RSD%) and the intraday repeatability 3.3 (80 mg kg^−1^ fat), 5.4 (400 mg kg^−1^ fat) and 2.5 (1000 mg kg^−1^ fat) RSD% (Imperiale et al., 2021).

### 2.6 NMR measurements

All NMR experiments were carried out at 25 °C using a 600 MHz spectrometer (JNM-ECZ from JEOL Ltd., Tokyo, Japan), equipped with a room temperature “Royal” HFX/FGSQ probe. *Standard solutions.* Dihydrosterculic acid (DHSA) and Lactobacillic acid (LBA) standard stock solutions were prepared by dissolving given amounts of DHSA and LBA, respectively, in 0.7 mL of CDCl₃ (containing 0.03 *v*/v % TMS as an internal standard) to reach a concentration of 2.0 mg mL^−1^. All 1D ^1^H NMR experiments were performed using a slight in-house modification of the pulse sequence “qnmr_experiment” (JEOL Ltd.), with the introduction of ^13^C decoupling during signal acquisition to achieve elimination of ^13^C-satellite signals. Homonuclear decoupling was used to increase the SNR of the *cis*-methylene proton of DHSA (and LBA). This was achieved by introducing a selective RF field (^1^H RF_1_) at $\delta_{1}$ = 0.60 ppm, during acquisition time, as previously described by our group (Eltemur et al., 2023).

*Complex mixtures* were obtained by dissolving a variable amount of extracted M-, G-, or H-milk fat ($W_{\mathrm{fat}}$) varying from ∼100 to ∼400 mg (depending on the extraction yield) in $V_{0}$ = 0.7 mL of CDCl_3_. The mixture was then vortexed for 1 min at room temperature and centrifuged at maximum speed (15,770 ×g) for 30 min at 4 °C to remove any insoluble particles that could affect the NMR shim during measurements. 1D ^1^H NMR experiments on “complex mixtures” were acquired as described previously for “standard solutions”. The LOD of the CPFAs signal (on both standard solutions and complex mixtures) was empirically determined through visual evaluation, based on the recognizable shape of the signal from the *cis*-methylene proton of the cyclopropane ring at ∼ −0.33 ppm in either DHSA or LBA (SNR ∼ 2–3). The LOQ of DHSA or LBA was established for SNR ≥ 10, ensuring a unique definition of DHSA or LBA concentration in line with previous studies (Badilita et al., 2012; Eltemur et al., 2023; Lister, 2005). Experimental concentrations of CPFAs (as sum of DHSA and LBA), ${\left[\text{CPFAs}\right]}_{\mathrm{NMR}}$, in both standard solutions and complex mixtures, were expressed as mg kg^−1^ of extracted milk fat and were calculated as follows:

(1) $${\left[\text{CPFA}\right]}_{\mathrm{NMR}}={\mathrm{MW}}_{\text{CPFA}}∙\frac{A_{1H_{C}}}{A_{\mathrm{TMS}}}∙\frac{N_{\mathrm{TMS}}}{N_{1H_{C}}}∙\frac{\left[\mathrm{TMS}\right]}{{\mathrm{MW}}_{\mathrm{TMS}}}∙\frac{V_{0}}{V_{f}}∙\frac{1}{W_{\mathrm{fat}}\left(\mathrm{Kg}\right)}∙\frac{1}{S_{\mathrm{DEC}}}$$

where ${\mathrm{MW}}_{\text{CPFA}}$ and ${\mathrm{MW}}_{\mathrm{TMS}}$ are the molecular weight of either DHSA or LBA (296.49 g mol^−1^) and TMS (88.22 g mol^−1^), respectively; A and N are the integrated area and number of ^1^H of DHSA and TMS, respectively. $\left[\mathrm{TMS}\right]$ is the concentration of TMS (expressed as mg mL^−1^), $W_{\mathrm{fat}}$ is the amount of milk fat dissolved in CDCl_3_ in the NMR tube, $\frac{V_{0}}{V_{f}}$ is the dilution factor which takes into account the density ($d_{\mathrm{fat}}$ = 0.8974 g cm^−3^) of the extracted milk fat with $V_{f}=V_{0}+\frac{W_{\mathrm{fat}}}{d_{\mathrm{fat}}}\;$, and $S_{\mathrm{DEC}}$ is the corresponding slope (= 1.4) obtained from the correlation of concentrations obtained in the absence and in the presence of homonuclear decoupling as shown in Fig. S2. All samples were analyzed in triplicate at 25 °C with the following acquisition parameters: acquisition time (AQ) = 4 s, spectrum width (SW) = 13 ppm (with filter limit = 4), frequency offset = 3 ppm, 90° ^1^H pulse = 7.9 ms, receiver gain = 56, dummy scans = 4 and number of scans (NS) = 2000. Chemical shift assignments for DHSA and LBA were achieved by a combination of ^1^H—^13^C HSQC-TOCSY and ^1^H—^13^C HSQC experiments acquired with NS = 60 and 16, respectively (see Fig. S3). The former was acquired with a mixing time of 50 ms. Both experiments were obtained with 1024 and 512 points in the direct and indirect dimensions with 50 % Poisson-gap Non-Uniform Sampling (NUS) schedule. Spectra reconstruction was achieved using the routine available on JEOL Delta software.

### 2.7 Data processing and statistical analysis

Peak integration and correlation of relative abundances of chemical compounds obtained from GC–MS measurements were performed with Lab Solution software (Shimadzu, Kyoto, Japan). NMR spectra processing and peak integration was achieved using Delta NMR Data Processing Software (JEOL Ltd., Tokyo, Japan).

The relationship between CPFAs content in M-milk measured by GC–MS (independent variable) and that measured by NMR (dependent variable) was first investigated using a linear model accounting for the CPFAs content measured by GC–MS, the sampling period (considered to be a fixed factor) and their interaction. Fulfillment of the assumption for the analysis (normal distribution of residuals, variance homogeneity) was visually checked by means of diagnostic plots. As neither the sampling period nor its interaction with the CPFAs concentration measured by GC–MS were significant (Table S2), the analysis was further conducted with a simplified model accounting only for the CPFAs concentration measured by GC–MS.

CPFAs, DHSA, LBA and their ratio R (DHSA/LBA) were analyzed using ANOVA with a model accounting for milk type, sampling period (both considered fixed factors), and their interaction. An appropriate transformation of the dependent variable (y_Ti_ = y_i_^-0.5^, where y_i_ is the original value and y_Ti_ is the transformed value) was applied for CPFAs and LBA (Table S3, Table S4) to meet the ANOVA assumptions. As no significant interaction between milk type and sampling period was detected, the analysis was repeated with a reduced model accounting for the milk type only. If a significant effect of milk type was detected, multiple comparisons were performed using Bonferroni-adjusted post-hoc tests (Salkind, 2010).

As the assumptions for the ANOVA could not be met for DHSA and R even after data transformation, the analysis was conducted using the non-parametric Kruskal-Wallis-test (Kruskal & Wallis, 1952), considering each combination of milk type and sampling period as a factor level (Table S5). When a significant effect of the investigated factor was found, post-hoc multiple comparisons were performed using the Conover-Iman test, with a Bonferroni correction applied (Conover, 1999). As within each milk type no significant differences between the sampling periods were detected, the analysis was repeated accounting for the milk type alone. The analysis of the relationship between DHSA and LBA was conducted as previously described for the relationship between [CPFAs]_GC__–__MS_ and [CPFAs]_NMR_.

*p*-values <0.05 were considered statistically significant. All formal analyses were performed with IBM SPSS Statistics (Version 29.0.1.0) except for the multiple comparisons according to Conover-Iman, for which the *conover.test* package in *R* (version 4.4.1) was used (Ihaka & Gentleman, 1996).

## 3 Results and discussion

**Fig. 1:**
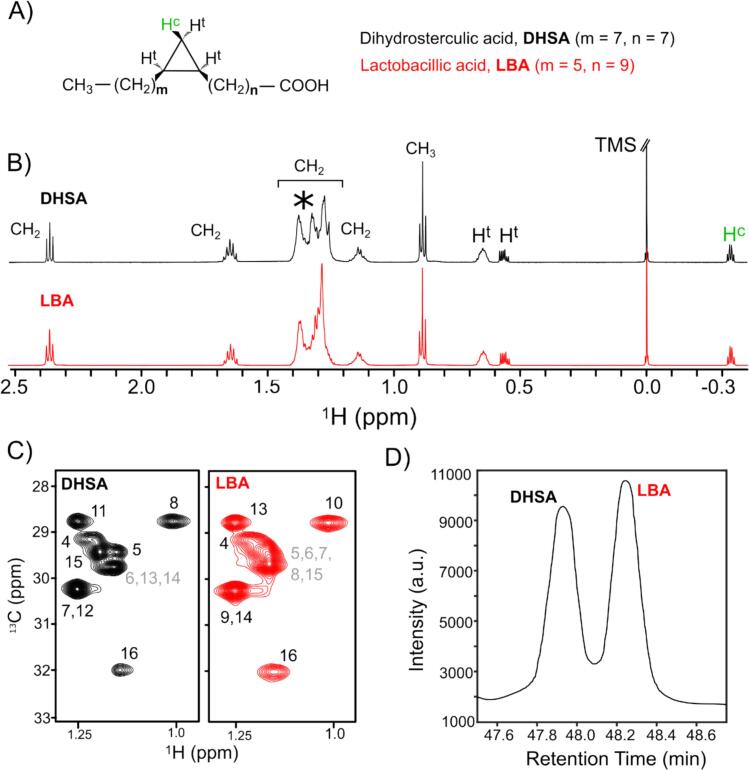
Spectral differentiation of DHSA and LBA by NMR spectroscopy and GC–MS. **(A)** Chemical structure of DHSA and LBA with protons of the cyclopropane ring highlighted as *cis* and *trans* by the labels H^c^ and H^t^, respectively. **(B)**^1^H NMR spectra of DHSA (black) and LBA (red) in the region from −0.4 to 2.5 ppm obtained in CDCl_3_, 600 MHz, 25 °C. The CH_2_ and CH_3_ groups are indicated in the spectra, with * highlighting the main differences. **(C)** Portion of ^1^H—^13^C HSQC spectra of DHSA (black) and LBA (red) with the corresponding assignment (in black) and overlapping (in gray). **(D)** GC–MS chromatogram obtained from a mixture of DHSA and LBA. (For interpretation of the references to colour in this figure legend, the reader is referred to the web version of this article.)

Dihydrosterculic acid (DHSA) and lactobacillic acid (LBA) are both fatty acids with an 18‑carbon chain. In DHSA, the cyclopropane ring is positioned between the 9th and 10th carbon atoms, whereas in LBA, it is located between the 11th and 12th carbon atoms (Fig. 1A). The 1D ^1^H NMR spectra of DHSA and LBA standard solutions in the region between −0.4 and 2.5 ppm are shown in Fig. 1B. Both spectra show an upfield quartet signal at ∼ −0.33 ppm which can be attributed to the *cis*-methylene proton of the cyclopropane ring (labelled as H^c^ in green in Fig. 1A). This signal is generally chosen as the target signal for quantification of CPFAs in NMR spectroscopy, given its distinct chemical shift far from any other resonances even in complex mixtures (Eltemur et al., 2023; Iannone et al., 2024; Knothe, 2006; Lolli et al., 2018). Downfield peaks at ∼0.55 ppm and ∼ 0.64 ppm can be assigned to *trans*-methylene proton, and to the two *trans*-methine protons of the cyclopropane ring (labelled as H^t^ in Fig. 1A), respectively. The downfield signals between ∼0.88 and ∼ 2.33 ppm can be assigned to the methylene and methyl protons of the fatty acid chain of both CPFAs. Although the chemical shifts in the NMR spectrum are highly sensitive to the electronic environment around the nuclei, the position of the cyclopropane ring along the carbon chain led to minimal differences in the ^1^H NMR spectra. In fact, only the distribution of methylene proton signals in the region 1.2–1.4 ppm appears to be affected, showing a different pattern of peaks when DHSA is compared to LBA (highlighted with * in Fig. 1B). To clarify this matter, we employed 2D ^1^H—^13^C HSQC experiments, which enhance resolution by correlating proton and carbon signals of the fatty acids. The overlapping 1D signals in the 1.2–1.4 ppm region were almost completely resolved in the 2D experiment, as shown in Fig. 1C, where the corresponding regions of methylene protons of DHSA (black) and LBA (red) are highlighted. The full 2D ^1^H—^13^C HSQC spectra are provided in the supplementary information (Fig. S3). ^1^H—^13^C HSQC-TOCSY experiments allowed for (almost) complete assignment of the chemical shifts in DHSA (except for H14). Distinct chemical shifts are observed for methylene protons which are one-bond (H8 and H11) and two-bonds (H7 and H12) away from the two *trans*-methine protons (H9 and H10) of the cyclopropane ring. However, the assignment of chemical shifts for methylene protons that are three- and four-bonds away from the cyclopropane rings (H6, H13, and H14, highlighted in gray in Fig. 1C) remains challenging, especially in the direction of the methyl group. Even greater difficulties were faced in the chemical shift assignments of methylene protons in the LBA spectra. The two-bond shift of the cyclopropane ring towards the -CH_3_ group creates an extended aliphatic region, resulting in significant signal overlap. This shift causes the overlapping of signals and increases the complexity of the chemical environment. Consequently, it becomes harder to distinguish and accurately assign the methylene proton signals. For LBA, only methylene protons directly bonded to the cyclopropane ring (H10 and H13) or positioned two bonds away (H9 and H14) could be assigned, as shown in Fig. 1C. Note that for both DHSA and LBA, the assignment of H16 is straightforward given its proximity to the terminal -CH_3_ group.

Given these challenges in resolving CPFAs by NMR alone (both in standard solutions and even more so in complex matrices), we opted to complement our NMR analysis with Gas Chromatography-Mass Spectrometry (GC–MS), a robust technique for identifying and characterizing both individual molecules and complex mixtures. Building on the experimental conditions detailed by Montanari et al. (2010), we enhanced the method by refining our temperature modulation strategy in GC as described in the Material and Methods. Fig. 1D illustrates the chromatogram obtained upon injection of methyl-esterified DHSA and LBA standard solutions. Two distinct peaks were observed in the chromatogram at retention times (RT) of 47.9 min and 48.3 min, corresponding to DHSA and LBA, respectively.

Once the identification of the two compounds could be achieved by NMR and GC–MS in standard solutions, we questioned whether individual detection and quantification of DHSA and LBA could be performed by these two techniques on complex matrices, i.e., milk samples from cows fed with diets excluding fermented feed or including maize or grass silage and no other fermented feed.

**Fig. 2:**
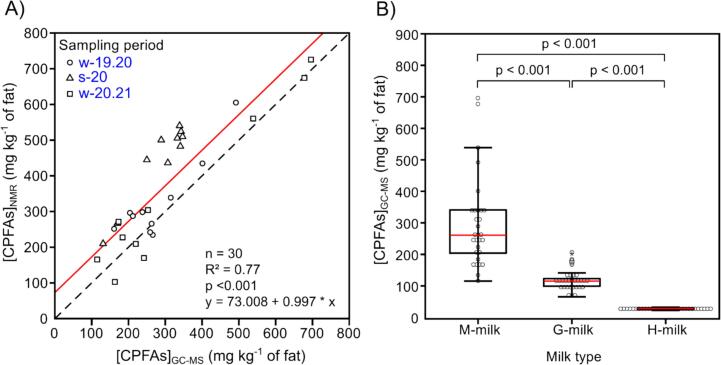
Quantification of CPFAs (as sum of DHSA and LBA) in complex mixtures. (A) Relationship of CPFAs (= [DHSA] + [LBA]) in M-milk as obtained from NMR ([CPFAs]_NMR_) and GC–MS analysis ([CPFAs]_GC__–__MS_). The regression line is shown in red colour, the black dashed line shows the 1:1 relationship; n = number of observations, R^2^ = coefficient of determination, p = probability value. (B) Boxplots comparing [CPFAs]_GC__–__MS_ in M-milk, G-milk and H-milk samples. The boxplots display the median (red line), interquartile range and outliers (observations not included between the whiskers). The *p*-values of post-hoc multiple comparisons according to Bonferroni following an ANOVA with transformed dependent variable (y_Ti_ = y_i_^-0.5^, where y_i_ is the original value and y_Ti_ is the transformed value) are displayed. (For interpretation of the references to colour in this figure legend, the reader is referred to the web version of this article.)

Quantification of CPFAs (as sum of the content of DHSA and LBA) through NMR spectroscopy was achieved by integrating the peak area corresponding to *cis*-methylene proton signal, H^c^ (centered approximately at −0.34 ppm) from 1D ^1^H experiments using the methyl signal of TMS as the internal standard. As previously described by our group, collapsing of DHSA signal quartet into a singlet through selective homonuclear decoupling improves the sensitivity of the DHSA signal and therefore facilitates accurate quantification. A quick comparison of peak heights estimated from the *cis*-methylene proton of DHSA in the presence and absence of a single decoupling field (RF_1_) shows a ∼ 1.5-fold increase in intensity (Fig. S4, right inset). To note, since no chemical shift differences were observed for the *cis*-methylene protons in the cyclopropane rings of either DHSA or LBA (see previous section), the integration of this signal allows for the quantification of [CPFAs] = [DHSA] + [LBA]. Analogously, quantification of CPFAs through GC–MS was achieved by summing up the amount of DHSA and LBA. Table S6 and Fig. 2B report the CPFAs content in the analyzed M-milk and G-milk samples (and H-milk as reference), categorized by the type of feed rations (containing either maize or grass silage or no silage). Both also include a comparison of CPFAs measurements obtained using NMR and GC–MS methods.

As expected, a moderately close linear relationship (R^2^ = 0.77) exists between ${\left[\text{CPFAs}\right]}_{\mathrm{NMR}}$ and ${\left[\text{CPFAs}\right]}_{\mathrm{GC}-\mathrm{MS}}$ obtained from ^1^H NMR spectra and GC–MS analysis, respectively, of M-milks from the three sampling periods (Fig. 2A). We tentatively attribute the dispersion along the regression line as well as the shift of the intercept to variations in sample preparation methods between NMR and GC–MS analyses. This discrepancy underscores the benefit of comparing both techniques, as it highlights how different analytical approaches can influence results and provides a more comprehensive understanding of the analytes' concentrations. By utilizing both NMR and GC–MS, measurements can be cross-validated, methodological differences accounted for, and a more accurate and reliable analysis of the compounds of interest achieved.

The content of CPFAs in M-milks and G-milks is comparable across the three sampling periods, with average values of ∼300 mg kg^−1^ and ∼ 100 mg kg^−1^ of fat, respectively (see Table S6). As shown in Fig. 2B, CPFAs concentrations in M-milk are consistently higher than those in G-milk, and both are significantly higher than those of H-milk. The observed difference between M-milk and G-milk is in line with previous research, suggesting that the lower availability of carbohydrates during grass fermentation may reduce the stress response of lactic acid bacteria (LAB). (Caligiani et al., 2016; Fava et al., 2025) Consequently, the lower stress could result in lower CPFAs levels in milk produced from diets that include grass silage rather than maize silage. Furthermore, as previously reported (Fava et al., 2025), the amount of CPFAs released daily in milk per cow is, on average, much higher than the respective daily CPFA intake. This outbound/inbound CPFA ratio is particularly elevated in M-milk compared to G-milk, especially in high starch intake diets like the one exclusively including maize silage. Notably, CPFA content has been reported to be negatively correlated with the levels of monounsaturated fatty acids (MUFAs), which are primarily associated with low starch intake (Lolli et al., 2021b).

**Fig. 3:**
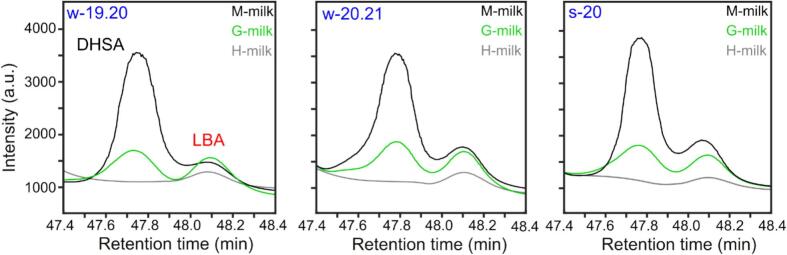
Overlap of GC–MS chromatograms for M-milk (black), G-milk (green), and H-milk (gray) samples across the three sampling periods (w-19.20, w-20.21, and s-20). Elution peaks corresponding to DHSA and LBA are shown in black and red, respectively. (For interpretation of the references to colour in this figure legend, the reader is referred to the web version of this article.)

Given the ability of GC–MS to precisely separate fatty acids like DHSA and LBA, we wonder if this method could provide accurate quantification of each compound in milk samples. Previous studies have generally focused on the cumulative quantification of CPFAs in milk without distinguishing between DHSA and LBA (Caligiani et al., 2014; Fava et al., 2025; Iannone et al., 2024; Imperiale et al., 2021; Marseglia et al., 2013). However, separating these acids using GC–MS may be of relevance in adding further details to CPFA presence in milk and milk derivatives, since LBA and DHSA originate from different metabolic pathways. Fig. 3 shows the GC–MS chromatographic separation achieved for DHSA and LBA in a subset of three milk samples (M-milk, G-milk, and H-milk) across the three sampling periods. It is immediately evident that the overall content of DHSA and LBA strongly depends on the feeding system (as shown in Fig. 2B). In M-milk, DHSA is the most abundant CPFA (shown in black in Fig. 3), whereas in G-milk, the contents of DHSA and LBA are almost equivalent (shown in green in Fig. 3).

A possible explanation for the different amounts of DHSA and LBA in M-milk and G-milk may lie in the response of lactic acid bacteria (LAB) to different stress conditions found in grass and maize silage. It has been reported that exposure to acid stress leads to an increase in the synthesis of C18:1n11 (vaccenic acid), a precursor of LBA (Montanari et al., 2010). This response is particularly pronounced under anaerobic conditions, suggesting that bacteria adapt to acidic environments by enhancing the production of this fatty acid. Conversely, the synthesis of C18:1n9 (oleic acid), a precursor of DHSA, is notably increased in response to low-temperature conditions (cold stress) (Montanari et al., 2010). This indicates that cold stress triggers the aerobic biosynthetic pathway in LAB, leading to higher levels of C18:1n9 as the organism adjusts to the stressor. The weak relationship (R^2^ = 0.35) between the concentration of DHSA and LBA measured in all milk samples considered in this study (Fig. S5) seems to support the different metabolic origins of the two CPFAs. However, a systematic study examining the presence and synthesis of C18:1 n11 and C18:1 n9 (and thereby LBA and DHSA) in *Lactobacillus* strains found in silage (mostly composed of homofermentative *L. plantarum* and heterofermentative species like L. *buchneri* and *L. brevis* (Caligiani et al., 2014; Rossi & Dellaglio, 2007) is necessary.

Unexpectedly, all H-milk samples contained LBA. Two possible routes for its presence in milk from cows fed exclusively with hay can be tentatively hypothesized: (i) LBA originates directly from the unensiled forage, or (ii) it is synthesized during digestion, which in ruminants involves passage through four stomachs (rumen, reticulum, omasum and abomasum) before reaching the intestine. With regard to the presence of CPFA (in particular LBA) in grass, it should be noted that plants recognized as possible sources of CPFA are usually not occurring in the grasslands of the area under study (Fava et al., 2025). It should also be noted that analysis of the fat extract of grass prior to fermentation/ensiling in Martini-Lösch et al. (2025) gave negative results. Therefore, it seems unlikely that LBA directly originates from the unensiled forage.

With reference to the possible synthesis of LBA during digestion, the CPFA presence or formation has been already investigated by simulating the rumen conditions in vitro (Lolli et al., 2021a). These results suggest that the rumen activity does not affect the CPFAs content. However, no studies concerning further digestion steps (reticulum, omasum and abomasum) with regard to CPFA have been reported in literature to the best of our knowledge. Moreover, LBA has been reported to be produced by specific intestinal bacteria as *Lactobacillus reuteri* (Jones et al., 2011; Lolli et al., 2020) making it possible to observe a baseline level of this compound due to their production and subsequent absorption at colonic level (Quach et al., 2019).

**Fig. 4:**
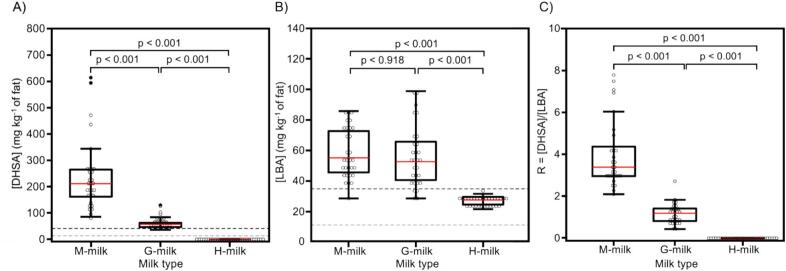
Boxplots comparing (A) DHSA, (B) LBA levels, as well as (C) their experimental ratio R (= [DHSA]/[LBA]) in M-milk, H-milk, and G-milk as obtained by GC–MS. The boxplots display the median (red line), interquartile range and outliers (observations not included between the whiskers). The *p*-values of post-hoc multiple comparisons according to Conover-Iman with a Bonferroni correction following a Kruskal-Wallis-test are shown for DHSA and R, while multiple comparisons according to Bonferroni following an ANOVA with data transformation (y_Ti_ = y_i_^-0.5^, where y_i_ is the original value and y_Ti_ is the transformed value) are shown for LBA. For DHSA and LBA, the gray dashed lines represent the respective limit of detection, the black dashed lines the respective limit of quantification. (For interpretation of the references to colour in this figure legend, the reader is referred to the web version of this article.)

The effect of the ration type on DHSA and LBA in milk is illustrated in the boxplots in Fig. 4A and Fig. 4B, providing insight into the differences in DHSA and LBA levels between the milk types. As shown in Fig. 4A, the multiple comparisons revealed a significant difference in DHSA levels between M-milk, G-milk and H-milk (*p* < 0.0001), suggesting that DHSA can be used as a differentiating factor. Conversely, as shown in Fig. 4B, the post-hoc test did not find a significant difference in LBA levels between G-milk and M-milk (*p* = 0.918), while both had significantly higher LBA levels than H-milk (both comparisons *p* < 0.001). This indicates that LBA may not be an effective differentiator between M-milk and G-milk.

We noted that the GC–MS chromatograms for H-milk samples show small quantities of LBA (Fig. 3A), generally ∼2-fold lower than in M- and G-milk samples (Table S6). However, no relevant amount of DHSA was measured in H-milk samples. To our knowledge, a systematic presence of LBA in H-milk has never been reported before. As expected, given the amount of [DHSA] ≤LOD (Fig. 4A) and [LBA] < LOQ (Fig. 4B) in H-milk samples, the Kruskal-Wallis test showed significant differences in both DHSA and LBA levels between H-milk and either M- or G- milk samples (p < 0.0001). The different content of DHSA and LBA is summarized in Fig. 4, where the ratio (*R*) of the concentrations of DHSA to LBA (*R* = [DHSA]/[LBA]), is represented through box plots. The average values of R obtained for M-milk and G-milk are approximately 3–4 and 1, respectively, while all H-milk values are zero.

## 4 Conclusion

In conclusion, our study demonstrated the potential of NMR spectroscopy and GC–MS for the identification and quantification of CPFAs in milk. This dual approach highlights the complementary strengths of each technique: NMR enables rapid screening with minimal sample preparation, whereas the refined GC–MS method provides accurate quantification and precise discrimination of individual CPFAs. A moderately close linear relationship between NMR- and GC–MS–derived CPFA concentrations suggests that NMR could serve as a quick alternative for CPFA quantification. NMR spectroscopy is indeed advantageous due to its minimal sample preparation and rapid analysis time.

Within the investigated pool of samples, the refined GC–MS method enabled precise separation and quantification of DHSA and LBA. DHSA emerged as the main CPFA differentiating M-milk and G-milk, while LBA levels did not differ between milk types. Therefore DHSA or the DHSA/LBA ratio also proved to be reliable markers. These results support the hypothesis that distinct metabolic pathways underlie variations in DHSA and LBA, which merits further microbial investigation. Notably, we report the unexpected presence of LBA in milk from cows having fed no fermented feeds (H-milk). This novel finding raises new questions about the metabolic origins and potential functional implications of LBA. Overall, this study underscores the value of multiple analytical techniques to enhance the accuracy and reliability of CPFAs quantification in complex matrices, paving the way for more detailed nutritional and metabolic studies in dairy science.

## CRediT authorship contribution statement

**Gian Marco Riccio:** Methodology, Investigation, Formal analysis, Data curation, Conceptualization. **Dilek Eltemur:** Investigation, Formal analysis, Data curation, Conceptualization. **Federico Fava:** Investigation, Formal analysis, Data curation, Conceptualization. **Demian Martini-Lösch:** Methodology, Investigation, Data curation. **Giovanni Peratoner:** Writing – review & editing, Visualization, Validation, Software, Methodology, Investigation, Formal analysis, Data curation, Conceptualization. **Elena Venir:** Writing – review & editing, Investigation, Data curation. **Daniela Eisenstecken:** Investigation. **Peter Robatscher:** Writing – review & editing, Methodology, Investigation, Formal analysis. **Matteo Scampicchio:** Supervision, Investigation, Funding acquisition. **Michael Oberhuber:** Writing – review & editing, Supervision, Funding acquisition. **Alberto Ceccon:** Writing – review & editing, Writing – original draft, Visualization, Validation, Supervision, Software, Resources, Project administration, Methodology, Investigation, Funding acquisition, Formal analysis, Data curation, Conceptualization.

## Funding

This research is funded by the FESR-EFRE 2014–2020 “Investitionen in Wachstum und Beschäftigung”, project Heumilch, FESR1129 CUP: H36H19000000007 and by the Autonomous Province of Bozen-Bolzano (Action plan for mountain agriculture, Decision n. 1016, 1 September 2015; NOI Capacity building I and II funding frame, Decision n. 1472, 7 Octobre 2013, Decision 864, 04 September 2018). Article publishing charge (APC) is covered by the Autonomous Province of Bozen-Bolzano.

## Declaration of competing interest

The authors declare that they have no known competing financial interests or personal relationships that could have appeared to influence the work reported in this paper.

## Data Availability

Data will be made available on request.

## Appendix A Supplementary data

Supplementary material**:** Figures of calibration curves for [CPFAs] using GC–MS, correlation plots of [CPFAs] determined from NMR in the presence and absence of decoupling pulses, 1D and 2D NMR spectra of DHSA and LBA, and the relationship between [DHSA] and [LBA] in M-, G-, and H-milk samples. Tables containing the concentrations of CPFAs (as the sum of DHSA and LBA) in milk samples obtained via GC–MS and ^1^H NMR measurements, along with tables useful for GC–MS and details about the results of the statistical analyses. Image 1
