# Supplementary material for: Differentiating cyclopropane fatty acids to support milk authenticity through GC–MS and NMR spectroscopy
Source: Food Chem X. 2025 Sep 16;31:103033. doi: 10.1016/j.fochx.2025.103033 (PMC12483655; doi:10.1016/j.fochx.2025.103033)
Supplement: Supplementary file 1 — Supplementary material: Figures of calibration curves for [CPFAs] using GC–MS, correlation plots of [CPFAs] determined from NMR in the presence and absence of decoupling pulses, 1D and 2D NMR spectra of DHSA and LBA, and the relationship between [DHSA] and [LBA] in M-, G-, and H-milk samples. Tables containing the concentrations of CPFAs (as the sum of DHSA and LBA) in milk samples obtained via GC–MS and 1H NMR measurements, along with tables useful for GC–MS and details about the results of the statistical analyses. [file mmc1.docx]

**Differentiating Cyclopropane Fatty Acids to support milk authenticity through GC-MS and NMR Spectroscopy**

Gian Marco Riccio^1^, Dilek Eltemur^2^, Federico Fava^1^, Demian Martini-Lösch^1^, Giovanni Peratoner^1^, Elena Venir^1^, Daniela Eisenstecken^1^, Peter Robatscher^1^, Matteo Scampicchio^2^, Michael Oberhuber^1^, and Alberto Ceccon^1,*^

1. Laimburg Research Centre, Laimburg 6 - Pfatten (Vadena), 39040 Auer (Ora), BZ, Italy.
2. Faculty of Agricultural, Environmental and Food Sciences, Free University of Bozen-Bolzano, Piazza Università 5, 39100 Bozen-Bolzano, Italy.

**Supplementary Materials**

**Table S1.** List of ions used as quantifiers and qualifiers for the detection and quantification of CPFAs. The m/z values for each compound were selected based on their intensity and the specificity of the fragmentation.

| **Compound** | **Quantifier (m/z)** | **Qualifier 1 (m/z)** | **Qualifier 2 (m/z)** |
| --- | --- | --- | --- |
| (±)-cis-9,10-methyleneoctadecanoic acid (DHSA) | 55 | 69 | 278 |
| (±)-cis-11,12-Methyleneoctadecanoic acid (LBA) | 55 | 69 | 278 |
| Acetophenone-d8 (IS) | 110 | 82 | 128 |

**Table S2.** Results of the formal analysis of the relationship of CPFA (= [DHSA] + [LBA]) in M-milk as obtained from GC-MS analysis ([CPFA]_GC-MS_) and NMR ([CPFA]_NMR_) by means of linear models. The full model accounts for [CPFA]_GC-MS_, the sampling period (Winter 2019-2020, Summer 2020 and Winter 2020-2021) and its interaction with [CPFA]_GC-MS_, while the reduced model accounts for [CPFA]_GC-MS_ only. Statistically significant terms are highlighted in bold. df = degrees of freedom, F = Fisher’s F, p = probability, - = not included in the model

| **Source** | Full model | | |  | Reduced model | | | | |
| --- | --- | --- | --- | --- | --- | --- | --- | --- | --- |
|  | df | F | p | | |  | df | F | p |
| [CPFA]_GC-MS_ | 1 | 156.02 | **<0.001** | | |  | 1 | 93.86 | **<0.001** |
| Sampling period (SP) | 2 | 0.10 | 0.909 | | |  | - | - | - |
| [CPFA]_GC-MS_*SP | 2 | 1.68 | 0.208 | | |  | - | - | - |
| Error | 24 |  |  | | |  | 28 |  |  |

**Table S3.** Results of ANOVA investigating the effect of the milk type (M-milk, G-milk and H-milk) on the content of CPFA (= [DHSA] + [LBA]) as obtained from GC-MS analysis. Model 1 accounts for milk type, sampling period (Winter 2019-2020, Summer 2020 and Winter 2020-2021) and their interaction, while Model 2 does not take the sampling period into account. The analyses were conducted after transformation of the dependent variable (y_Ti_= y_i_^-0.5^, where y_i_ is the original value and y_Ti_ is the transformed value). Statistically significant terms are highlighted in bold. df = degrees of freedom, F = Fisher’s F, p = probability.

| Source | CPFA | | | | | | |
| --- | --- | --- | --- | --- | --- | --- | --- |
|  | Full model | | |  | Reduced model | | |
|  | df | F | p |  | df | F | p |
| Milk type (MT) | 2 | 962,6 | **<0.001** |  | 2 | 933,1 | **<0.001** |
| Sampling period (SP) | 2 | 2,5 | 0,088 |  | - | - | - |
| MT*SP | 4 | 1,0 | 0,434 |  | - | - | - |
| Error | 80 |  |  |  | 86 |  |  |

**Table S4.** Results of ANOVA investigating the effect of the milk type (M-milk, G-milk and H-milk) on the content of LBA as obtained from GC-MS analysis. Model 1 accounts for milk type, sampling period (Winter 2019-2020, Summer 2020 and Winter 2020-2021) and their interaction, while Model 2 does not take the sampling period into account. The analyses were conducted after transformation of the dependent variable (y_Ti_= y_i_^-0.5^, where y_i_ is the original value and y_Ti_ is the transformed value). Statistically significant terms are highlighted in bold. df = degrees of freedom, F = Fisher’s F, p = probability.

| Source | LBA | | | | | | | | |
| --- | --- | --- | --- | --- | --- | --- | --- | --- | --- |
|  | Full model | | |  | | Reduced model | | | |
|  | df | F | p | |  | | df | F | p |
| Milk type (MT) | 2 | 110.9 | **<0.001** | |  | | 2 | 90.0 | **<0.001** |
| Sampling period (SP) | 2 | 9.7 | **<0.001** | |  | | - | - | - |
| MT*SP | 4 | 2.0 | 0.109 | |  | | - | - | - |
| Error | 80 |  |  | |  | | 88 |  |  |

**Table S5.** Results of Kruskal-Wallis tests investigating the effect of the milk type (M-milk, G-milk and H-milk) on the content of DHSA, the ratio of DHSA and LBA as obtained from GC-MS analysis. Model 1 treats each combination of milk type and sampling period (Winter 2019-2020, Summer 2020 and Winter 2020-2021) as separate levels, while Model 2 does not take the sampling period into account. Statistically significant terms are highlighted in bold. df = degrees of freedom, χ² = chi-square test statistics, p = probability.

| Dependent variable | Model 1 | | |  | Model 2 | | |
| --- | --- | --- | --- | --- | --- | --- | --- |
|  | χ² | df | p |  | χ² | df | p |
| [DHSA] | 80.76 | 8 | **<0.001** |  | 80.61 | 2 | **<0.001** |
| R = [DHSA]/[LBA] | 82.51 | 8 | **<0.001** |  | 80.88 | 2 | **<0.001** |

**Table S6**. Concentration of CPFAs (as sum of DHSA and LBA) in the milk samples obtained with GC-MS and ^1^H NMR measurements. Averages ± standard deviation were calculated from a milk sample set (n) of 10 for each sampling period except for G-milk in Winter 2019-2020 (n = 9). Note that 1 M-milk, 3 G-milk and all H-milk samples had <[LBA]> below the LOQ.

|  | **^1^H NMR^a^** | **GC-MS** | |
| --- | --- | --- | --- |
| Sampling period/ Feeding regime | <[CPFA]> (mg kg^-1^) | <[CPFA]> (mg kg^-1^) | $<R> = \frac{[DHSA]}{[LBA]}$ |
| *Winter 2019-2020* |  |  |  |
| Maize (M-milk) | 305 ± 67 | 281 ± 99 | 4.67 ± 0.47 |
| Grass (G-milk) | 92 ± 40 | 106 ± 33 | 1.48 ± 0.18 |
| Hay (H-milk) | < LOD | 25 ± 3 | 0.00 ± 0.00 |
| *Winter 2020-2021* |  |  |  |
| Maize (M-milk) | 321 ± 50 | 326 ± 222 | 4.14 ± 0.67 |
| Grass (G-milk) | < LOD | 125 ± 32 | 0.89 ± 0.10 |
| Hay (H-milk) | < LOD | 29 ± 3 | 0.00 ± 0.00 |
| *Summer 2020* |  |  |  |
| Maize (M-milk) | 442 ± 70 | 285 ± 78 | 3.12 ± 0.40 |
| Grass (G-milk) | < LOD | 116 ± 26 | 1.16 ± 0.41 |
| Hay (H-milk) | < LOD | 28 ± 3 | 0.00 ± 0.00 |

^a^ Limit of quantification (LOQ) of CPFAs with NMR was set for Signal-to-Noise Ratio (SNR) = 10. All measurements with SNR < 10 were excluded from the analysis. ^b^ In H-milk samples <[CPFA]> = <[LBA]> since no DHSA was detected.


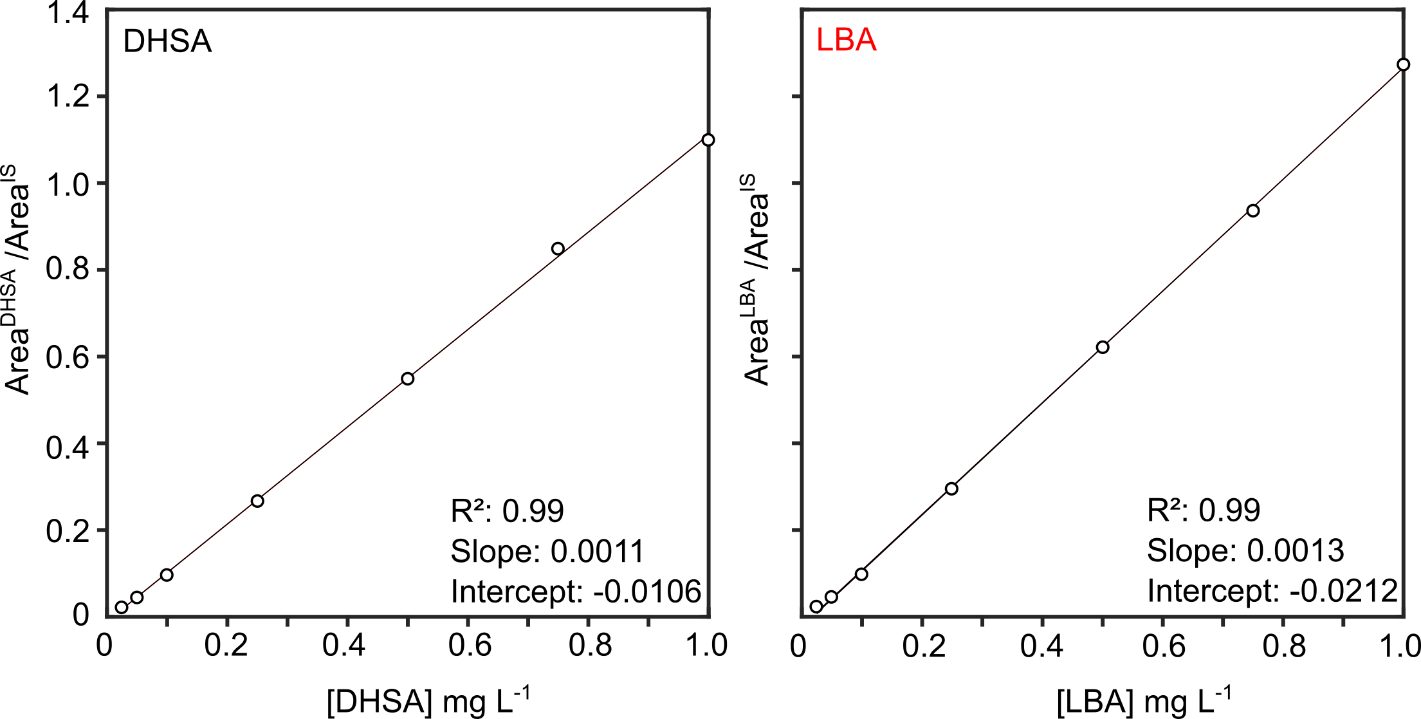


**Figure S1.** Calibration curves of DHSA (left) and LBA (right) in transesterified complex mixture using GC-MS. The area ratios were obtained from integration of the corresponding single ion (quantifier): 55 m/z for DHSA and LBA, and 110 m/z for Internal Standard (IS). These ratios were plotted against the concentration of either DHSA or LBA. Data points are shown as the average of five instrumental repetitions, with standard deviations smaller than the data points.


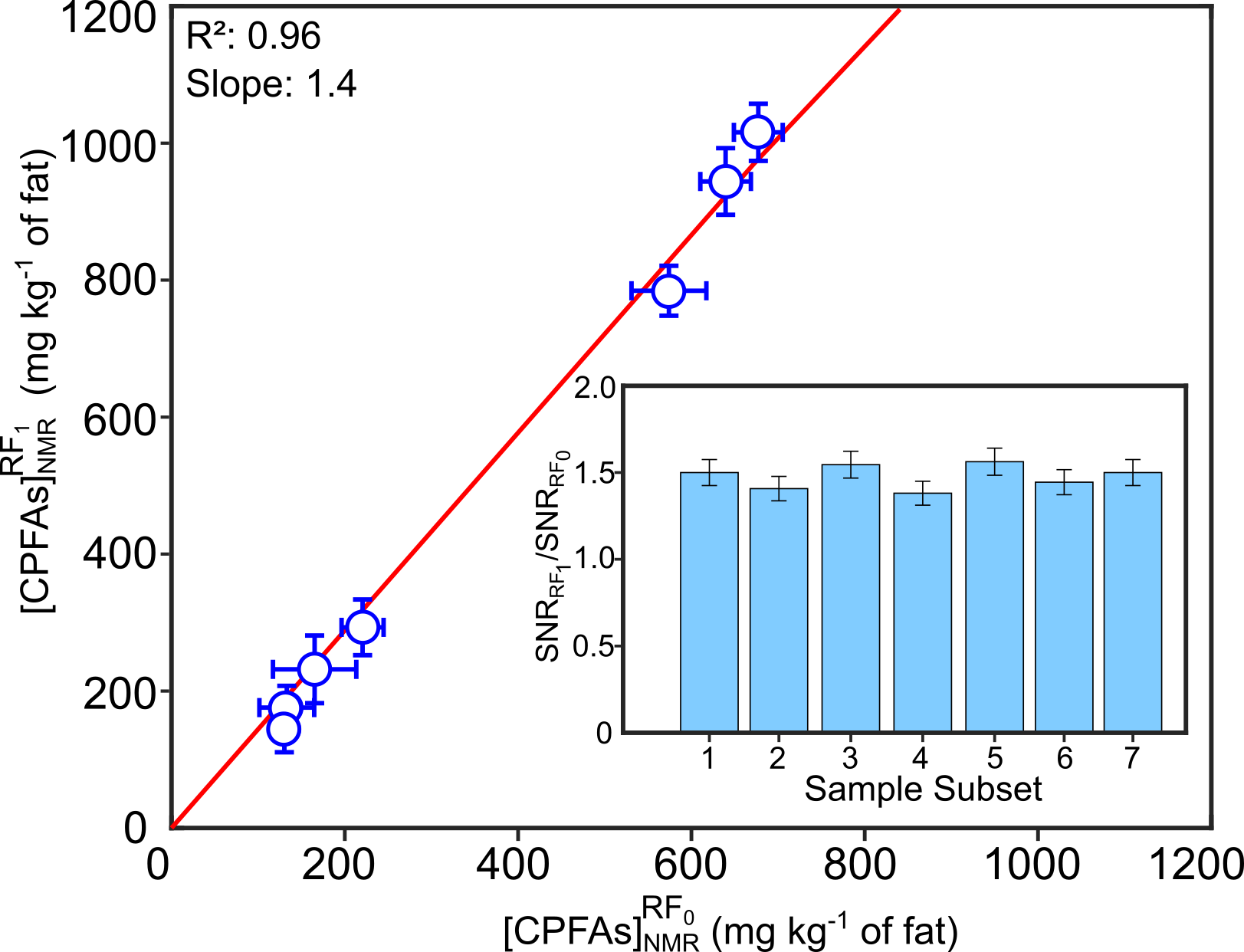


**Figure S2.** Correlation plot comparing the concentration of CPFAs (as sum of DHSA and LBA) determined from NMR experiments acquired without (RF_0_) (x-axis) or with single (RF_1_) selective decoupling pulses (y-axis). Both concentrations were determined from the integrated area of cis-methylene proton signal of the cyclopropane ring in ^1^H NMR spectra of complex mixtures. Inset: Bar graph of the experimental signal-to-noise (SNR) ratios (${{=SNR}_{RF1}}/{{SNR}_{{RF}_{0}})}$ estimated from cis-methylene proton signal of experiments acquired with single (RF_1_) or without (RF_0_) decoupling pulses. NMR signal and noise were extracted as described in the Material and Methods. NMR analyses were carried out in triplicate for each experiment and error bars are reported in the plots. All measurements were performed on a subset (n = 7) of samples obtained from cows fed with maize silage (M-milk) from the Winter 2020-2021 feeding period.


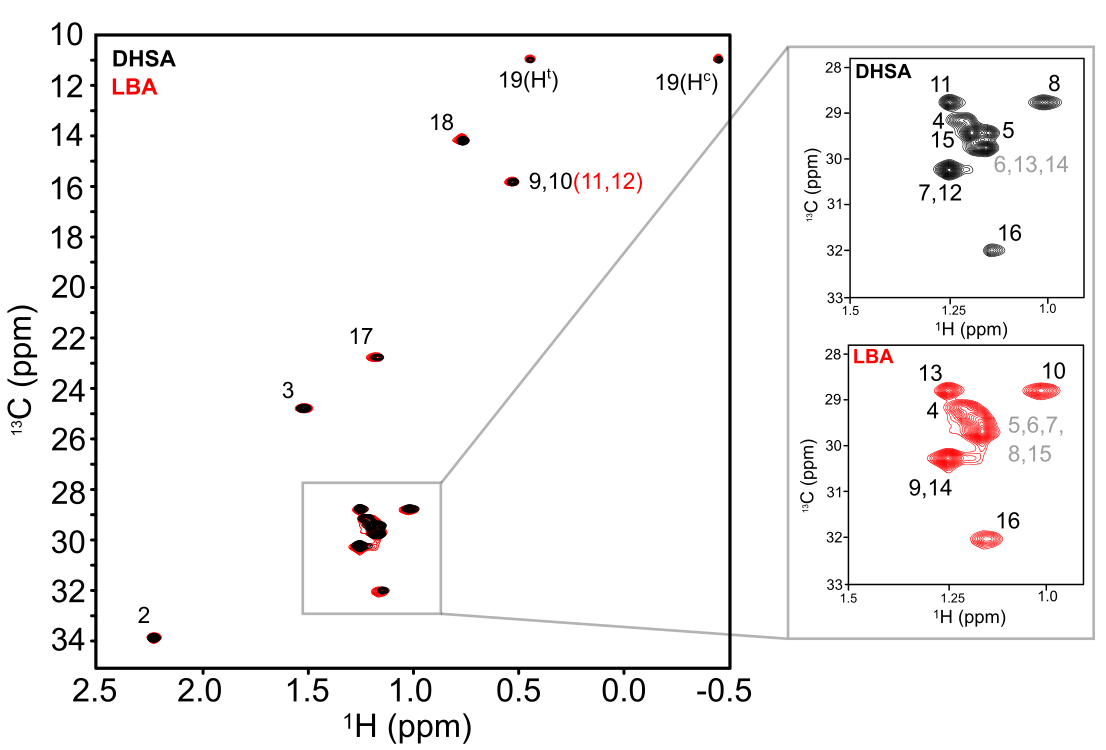


**Figure S3.** Overlay of ^1^H-^13^C HSQC spectra of DHSA (in black) and LBA (in red). The portion of the spectra enclosed in the gray box is zoomed in on the right side of the figure. Cross-peaks are labeled with the assigned signals in black, except for the signal corresponding to H11, H12, which accounts for different numbering in LBA respect to DHSA. Non-assignable atoms of both compounds due to overlapping signals are labeled in gray in the zoomed figures. The 2D spectra were obtained at 600 MHz and 25 °C.


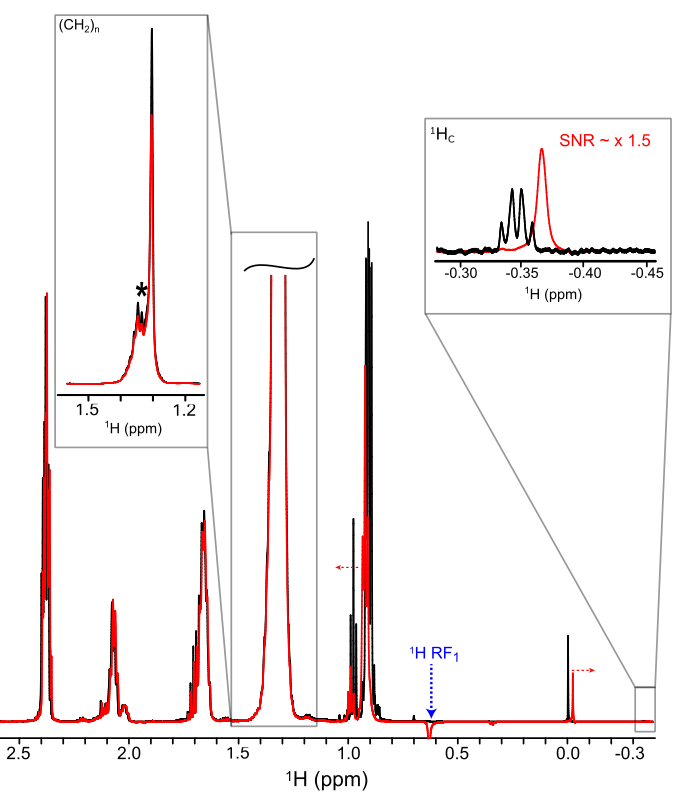


**Figure S4.** Overlay of representative 1D ^1^H spectra obtained on milk from maize silage feeding in the absence (black) and in presence (red) of the applied homonuclear decoupling, with $\omega^{{RF}_{1}}$ = 0.6 ppm (as highlighted with the blue labelled). Enlargement (on the left) shows the overlay of signal attributable to CH_2_ of both DHSA and LBA molecules. As shown in Figure 1B and 1C, the signal highlighted with * is attributable to (only) H15 of the DHSA acid. Enlargement (on the right) in the region from -0.3 to -0.45 ppm of the spectra shows the signal assigned to 1HC used for CPFA quantification in the studied milk samples. Improved detection of ${{}^{1}H}^{c}$ signal is evident upon removal of coupling and from the increase in the SNR factor (~ factor of ~ 1.5 as reported in Eltemur et al.^1^) All experiments were performed at 600 MHz and 25 °C in CDCl_3_. Correct comparison of all spectra was achieved by using the CHCl_3_ signal as a reference for both intensity and chemical shift. This reference is necessary because the application of the RF field induces Bloch–Siegert (BS) shifts on both the signal at approximately 0.9 ppm and the TMS signal (highlighted with red arrows)

**
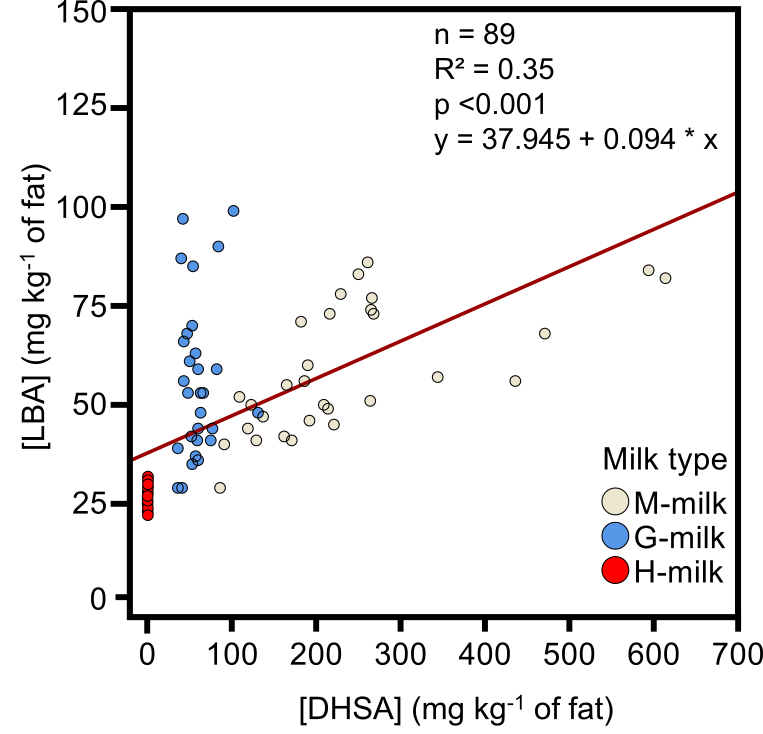
**

**Figure S5**. Relationship between LBA and DHSA in M-milk (brown dots), G-milk (blue dots) and H-milk (red dots) samples obtained by GC-MS. The regression line is shown in red color, n = number of observations, R² = coefficient of determination, p = probability value.
